# Prognostic value of cuproptosis-related genes signature and its impact on the reshaped immune microenvironment of glioma

**DOI:** 10.3389/fphar.2022.1016520

**Published:** 2022-10-04

**Authors:** Siliang Chen, Shuxin Zhang, Yunbo Yuan, Zhihao Wang, Junhong Li, Tengfei Li, Mingrong Zuo, Wentao Feng, Mina Chen, Yanhui Liu

**Affiliations:** ^1^ Department of Neurosurgery, West China Hospital of Sichuan University, Chengdu, Sichuan, China; ^2^ Department of Head and Neck Surgery, Sichuan Cancer Hospital and Institute, Sichuan Cancer Hospital, School of Medicine, University of Electronic Science and Technology of China, Chengdu, China; ^3^ State Key Laboratory of Biotherapy, Neuroscience & Metabolism Research, West China Hospital, Sichuan University, Chengdu, China

**Keywords:** cuproptosis, glioma, ion-dependent cell death, prognosis, immune infiltration

## Abstract

Glioma is the most prevalent malignancy in the central nervous system. The impact of ion-induced cell death on malignant tumors’ development and immune microenvironment has attracted broad attention in recent years. Cuproptosis is a novel copper-dependent mechanism that could potentially regulate tumor cell death by targeting mitochondria respiration. However, the role of cuproptosis in gliomas remains unclear. In the present study, we investigated the relationships between the expression of cuproptosis-related genes (CRGs) and tumor characteristics, including prognosis and microenvironment of glioma, by analyzing multiple public databases and our cohort. Consensus clustering based on the expression of twelve CRGs stratified the glioma patients into three subgroups with significantly different prognosis and immune microenvironment landscapes. Reduced immune infiltration was associated with the less aggressive CRG cluster. A prognostic CRGs risk signature (CRGRS), based on eight critical CRGs, classified the patients into low- and high-risk groups in the training set and was endorsed by validation sets from multiple cohorts. The high-risk group manifested a shorter overall survival, and further survival analysis demonstrated that the CRGRS was an independent prognostic factor. The nomogram combining CRGRS and other clinicopathological factors exhibited good accuracy in predicting the prognosis of glioma patients. Moreover, analyses of tumor immune microenvironment indicated that higher CRGRS was correlated with increased immune cell infiltration but diminished immune function. Gliomas in the high-risk group exhibited higher expression of multiple immune checkpoints, including PD-1 and PD-L1, and a better predicted therapy response to immune checkpoint inhibitors. In conclusion, our study elucidated the connections between CRGs expression and the aggressiveness of gliomas, and the application of CRGRS derived a new robust model for prognosis evaluation of glioma patients. The correlations between the profiles of CRGs expression and immune tumor microenvironment illuminated prospects and potential indications of immunotherapy for glioma.

## Introduction

Glioma is the most common malignant tumor in the central nervous system (CNS) and accounts for approximately 80% of all malignant CNS tumors (Ostrom et al., 2021). Surgery with adjuvant radiotherapy and chemotherapy comprises the current standard treatment regime (Stupp et al., 2005). Despite thorough therapy procedure, the overall survival (OS) for many glioma patients is still poor (Weller et al., 2021), especially glioblastoma, which is extremely malignant and manifests a median OS of fewer than 2 years (Chinot et al., 2014; Gilbert et al., 2014; Stupp et al., 2015). Hence, many studies experimented with novel therapies for glioblastoma, aiming to improve patient survival. Immunotherapy was proved effective in many other tumors (Zhang and Zhang, 2020). For example, immune-checkpoint inhibitors (ICIs) have demonstrated positive efficacy in treating melanoma (Larkin et al., 2015), non-small-cell lung cancer (NSCLC) (Reck et al., 2016), and cervical cancer (Tewari et al., 2022). In the CNS, the ICIs could prolong patient OS in metastatic melanoma (Tawbi et al., 2018) and NSCLC (Hendriks et al., 2019), indicating that ICIs can deliver robust anti-tumor effect in the immune microenvironment of CNS. Neoadjuvant ICIs were proved to modify the tumor immune microenvironment and immune response in glioblastoma (Cloughesy et al., 2019; Schalper et al., 2019). Nevertheless, several phase III trials of ICIs in patients with glioblastoma eventually achieved insignificant results (Reardon et al., 2020; Lim et al., 2022; Omuro et al., 2022). These failures suggested that further research is needed to understand the tumor microenvironment (TME) and immune landscape of gliomas and their influence on the effect of immunotherapy in glioma (Ott et al., 2021).

Cuproptosis, a newly discovered regulated cell death (RCD) type, was defined as copper-induced cell death (Tsvetkov et al., 2022). Copper has been recognized as a cofactor for several essential enzymes (Kim et al., 2008). Moreover, intracellular copper concentrations are regulated by a couple of active homeostatic mechanisms to prevent excessive accumulation of intracellular copper (Rae et al., 1999; Lutsenko, 2010). In lung cancer and breast cancer cells, copper could induce cell death by targeting the lipoylated tricarboxylic acid cycle. With the assistance of whole-genome CRISPR-Cas9 positive selection screen technology, seven genes were confirmed to be associated with resistance to cuproptosis (FDX1, LIAS, LIPT1, DLD, DLAT, PDHA1, and PDHB), and three genes were linked with the sensibility to cuproptosis (MTF1, GLS, and CDKN2A). Additionally, SLC31A1 (CTR1) and ATP7A/B, which encode the copper importer and exporter, showed a tight correlation with sensitivity to copper concentration (Tsvetkov et al., 2022). Interestingly, a previous *in vivo* PET study found enhanced localization of copper isotype tracer in the hypoxic areas of gliomas, coinciding with increased CTR1 expression (Pérès et al., 2019). Meanwhile, it is speculated that deprivation of copper by tumor cells from TME may impair copper-dependent SOD enzymes in immune cells, and convert tumor-associated macrophages (TAMs) into pro-tumoral M2 phenotype (Serra et al., 2020). Nonetheless, the role of cuproptosis in the development of gliomas and their TME was not well elucidated.

In our present study, multiple cohorts, including TCGA, CGGA, REMBRANDT, and our own patient cohort, were utilized to investigate the impact of cuproptosis-related gene expression on the characteristics of gliomas. We constructed a cuproptosis-related gene (CRG) signature to evaluate the clinical implications of CRG expression. Besides, we also evaluated and clarified the correlations between the CRG signature and the landscape of the glioma immune microenvironment.

## Materials and methods

### Data collection and preprocessing

RNA-seq data and clinical information of glioma patients were obtained from public databases and the REMBRANDT database. We downloaded fragments per kilobase million (FPKM) data of 662 primary gliomas from the Cancer Genome Atlas (TCGA, of which 655 had survival data). FPKM data of 226 primary gliomas, as well as the array data of 369 gliomas from the REMBRANDT (Gusev et al., 2018) cohort, were downloaded from the Chinese Glioma Genome Atlas (CGGA) curation (Zhao et al., 2021) (http://www.cgga.org.cn/). Genes expressed at a too low level (maximum FPKM <0.1) were excluded from the analysis.

Our cohort contained 77 primary glioma patients enrolled at West China Hospital (WCH). mRNA-sequencing data of their glioma tumor tissue obtained during craniotomy were quantified using STAR and normalized into FPKM. The survival data of these patients were acquired through telephone interviews every 3–6 months. Overall survival (OS) was defined as the period from surgery to death or the end of the last interview (censored value). Among all cohorts, patients with age <18 were excluded. Available clinicopathological information of all the four cohorts was shown in Table 1.

**TABLE 1:** Clinicopathological characteristics of adult primary glioma patients in TCGA, CCGA, REMBRANDT, and WCH cohort.

Characteristics	TCGA (N = 662)	CGGA (N = 226)	REMBRANDT (N = 369)	WCH (N = 77)
Age mean(range)	46 (18–89)	52 (22–87)	52 (22–87)	46 (19–77)
Gender				
Female	282 (42.6%)	87 (38.5%)	118 (32.0%)	30 (39.0%)
Male	380 (57.4%)	139 (61.5%)	196 (53.1%)	47 (77.0%)
NA	0	0	55 (14.9%)	0
Histology				
Astrocytoma	341 (51.5%)	82 (36.3%)	133 (36.0%)	22 (28.6%)
Oligodendroglioma	167 (25.2%)	60 (26.6%)	59 (16.0%)	21 (27.3%)
Glioblastoma	154 (23.3%)	84 (37.2%)	177 (48.0%)	34 (44.2%)
Grade				
G2	214 (32.3%)	94 (41.6%)	88 (23.8%)	29 (37.7%)
G3	237 (35.8%)	48 (21.2%)	66 (17.9%)	14 (18.2%)
G4	154 (23.3%)	84 (37.2%)	177 (48.0%)	34 (44.2%)
NA	57 (8.6%)	0	38 (10.3%)	0
IDH status				
Mutant	421 (63.6%)	115 (50.9%)	NA	42 (54.5%)
WT	236 (35.6%)	110 (48.7%)	NA	35 (45.5%)
NA	5 (0.8%)	1 (0.4%)	NA	0
1p19q Codeletion				
Codel	167 (25.2%)	54 (23.9%)	24 (6.5%)	19 (24.7%)
Non-codel	488 (73.7%)	169 (74.8%)	148 (40.1%)	43 (55.8%)
NA	7 (1.1%)	3 (1.3%)	197 (53.4%)	15 (19.5%)
TERT promoter status				
Mutant	340 (51.4%)	NA	NA	30 (39.0%)
WT	156 (23.6%)	NA	NA	23 (29.9%)
NA	166 (25.1%)	NA	NA	24 (31.2%)
MGMT promoter status				
Methylated	472 (71.3%)	97 (42.9%)	NA	35 (45.5%)
Unmethylated	157 (23.7%)	115 (50.9%)	NA	13 (16.9%)
NA	33 (5.0%)	14 (6.2%)	NA	29 (37.7%)
ATRX status				
Mutant	192 (29.0%)	NA	NA	22 (28.6%)
WT	459 (69.3%)	NA	NA	53 (68.8%)
NA	11 (1.7%)	NA	NA	2 (2.6%)

Abbreviation: TCGA, The Cancer Genome Atlas; CGGA, Chinese Glioma Genome Atlas; WCH, West China Hospital; IDH, isocitrate dehydrogenase; TERT, telomerase reverse transcriptase; MGMT, O6-methylguanine-DNA, methyltransferase; ATRX, alpha-thalassemia x-linked intellectual disability syndrome; WT, wild type; NA, not available.

### Unsupervised clustering analysis using CRGs

Twelve cuproptosis-related genes (CRGs), including LIAS, LIPT1, PDHB, GLS, PDHA1, ATP7B, CDKN2A, MTF1, FDX1, SLC31A1, DLAT, and DLD, were defined according to previous literature (Tsvetkov et al., 2022). Consensus clustering analysis was conducted to depict different cuproptosis patterns in glioma based on the expression level of CRGs using the R package “ConsensusClusterPlus” with 100 iterations (Wilkerson and Hayes, 2010). The cluster number was determined based on the cumulative distribution function (CDF) curve of the consensus index and the sample size. Through this process, we tried to expand the sample size of each cluster and keep a smoothly escalating CDF. To visualize the transcriptomic distinctions among all the clusters, we conducted the t-Distributed Stochastic Neighbor Embedding (tSNE) analysis with the expression of CRGs. After exploration of the CRG expression clusters, we then trained a naïve Bayes model using the CRG expression and cluster labels in the TCGA dataset and subsequently stratified samples in the other three cohorts.

### Construction and validation of cuproptosis-related gene risk signature

To investigate the correlation between CRG expression and glioma prognosis, we constructed a CRGs risk signature evaluation system. The TCGA dataset was first split into the training and test sets at a ratio of 6:4, while those from the other three datasets were held out as validation sets. In the training set, the CRGs were screened using least absolute shrinkage and selection operator (LASSO) Cox regression analysis. The genes whose coefficient was not 0 at the lambdas minimum C-index in 100 random repetitions of LASSO Cox regression were identified as critical CRGs in glioma, and the risk signature was constructed based on the expression of these critical CRGs. The cuproptosis-related genes risk signature (CRGRS) was calculated using the following formula:

$$CRGs\;Risk\;Signature=\underset{i=1}{\sum }\left({\beta_{i}*\mathrm{Exp}}_{i}\right)$$

In this formula, *β* and *Exp* stand for the coefficients and expression levels of each critical CRGs, respectively. All patients were allocated to high-risk or low-risk groups according to the optimal CRGRS cut-off value determined by ‘surv_cutpoint’ in the R package “survminer” with group proportion ≥0.1. Furthermore, we illustrated the receiver operating characteristic (ROC) curve in validation sets of 1/2/3-years survival and calculated the area under the ROC curve (AUC) using the R package “timeROC”.

### Somatic mutation and copy number variation analysis

To analyze different patterns of somatic mutations and copy number variations (CNVs) between consensus clusters and CRGRS-related risk groups, we obtained somatic mutations and CNVs data of patients in the TCGA cohort from the cBioPortal database (https://www.cbioportal.org). The most frequent gene mutations were visualized using the R package “maftools”. The Genomic Identification of Significant Targets in Cancer (GISTIC) score was used to evaluate the CNV levels.

### Functional enrichment analysis and tumor microenvironment immune landscape evaluation

To elucidate the differences of enrichment between different consensus clusters and CRGRS risk groups, over-representation and gene set enrichment analysis (GSEA) was used to assess differentially expressed genes (DEG) with Gene Ontology (GO) enrichment with the R package “clusterProfiler”. DEGs between groups were identified using R package ‘limma’ and were defined as those with adjusted *p*-value < 0.05 and |log_2_FC| > 0.5. The R package “GSVA” was used to transfer the logFPKM matrix to pathway expression matrix, and “limma” was used to identify the differentially expressed pathways among the clusters. The website CIBERSORTx (https://cibersortx.stanford.edu/) was used to calculate the absolute infiltration fraction of immune cells in glioma. Moreover, we utilized the Estimation of Stromal and Immune cells in Malignant Tumor tissues using Expression data (ESTIMATE) to estimate the infiltration of immune and stromal cells tumor microenvironment (Yoshihara et al., 2013). For tumor purity, we used the results published by D. Aran et al., which included tumor purity computed by the ESTIMATE algorithm and the consensus purity estimation (CPE) approach (Aran et al., 2015). In silico analysis of T cell exclusion, dysfunction in the TME, and prediction of ICI response were conducted using the TIDE suite (tide.dfci.harvard.edu) (Jiang et al., 2018).

### Prognostic factor analysis and nomogram construction

CRGRS and other potential prognostic factors, including the tumor grade, age, radiotherapy, chemotherapy, gender, KPS, 1p/19q codeletion, and IDH mutation, were included in the univariate Cox regression analysis. Subsequently, the factors confirmed as prognostic factors in univariate analysis (*p* < 0.05) were assessed in multivariate Cox regression analysis.

Those factors, confirmed potential independent prognostic factors, were united to construct a nomogram with the R package ‘rms’. Finally, the calibration curves and receiver operating characteristic (ROC) curves were used to evaluate the efficiency of the nomogram for predicting the prognosis of glioma patients.

### Statistical Analysis

The bioinformatic analyses were completed in the R software (version 3.6.1). For continuous variables, the Wilcoxon rank sum test was used to determine the difference between two groups, and Kruskal–Wallis one-way analysis of variance followed by post hoc Wilcoxon tests was conducted for three or more groups. For those categorical variables, the chi-square test was used to determine the difference in proportions. The R package ‘survminer’ was used to perform Kaplan-Meier (KM) analysis. The differences between KM curves were determined using the log-rank test. Cox regression analysis was conducted using the coxph function in R package “survival” and LASSO-Cox regression was performed using R package ‘glmnet’. To ensure the robustness of correlation analysis, we removed the outliers using iterative Grubbs test before fitting linear regression to scatter plots.

### Ethical approval and consent to participate

Tumor samples and clinical data collection and use were performed in strict accordance with ethics regulations and approved by the institutional review board of West China Hospital (No. 2018.569) based on local ethics regulations and the 1964 Helsinki declaration and its later amendments. The patients signed written consent for tumor tissue collection and processing.

## Results

### Consensus clustering analysis of CRG expression discovered three distinct glioma subgroups

**FIGURE 1:**
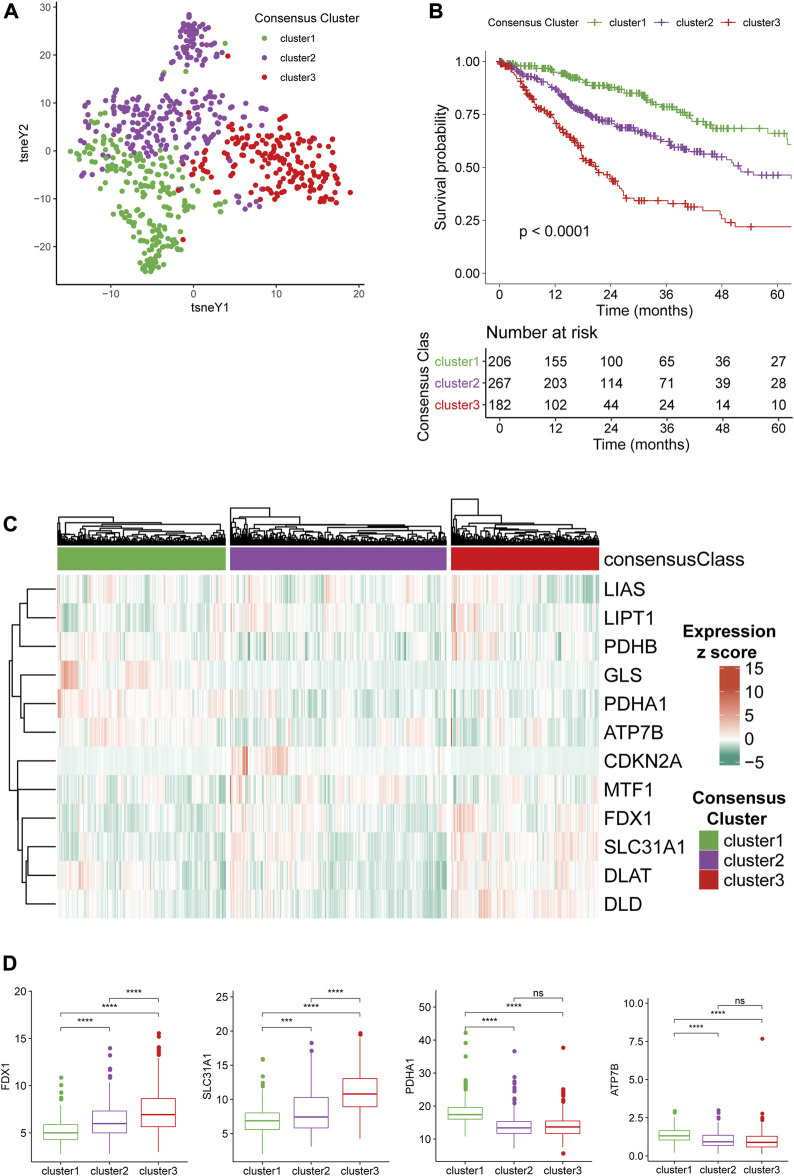
Consensus clustering of gliomas based on CRG expression. **(A)**CRG expression tSNE of the consensus clusters. **(B)** Kaplan-Meier Curve of the consensus clusters (*p* < 0.0001). **(C)** Heatmap of the expression level of 12 CRGs in the consensus clusters. **(D)** The expression level of FDX1, SLC31A1, PDHA1, and ATP7B between the consensus clusters.

To understand the expression patterns CRGs in gliomas, we conducted an unsupervised consensus clustering analysis of the TCGA glioma samples with the expression of the twelve CRGs. By evaluating the CDF and cluster size according to the principles described in Materials and Methods, we could classify them into four consensus clusters. However, the survival analysis demonstrated that two clusters (cluster1 and 2) showed highly similar CRG expression patterns and patient outcomes (Supplementary Figure S1). In the meantime, these two clusters were essential subsets from cluster 1 if the dataset was divided into three consensus clusters. Therefore, we merged these two clusters, eventually classifying the gliomas into three consensus clusters and verifying their distinction in CRG expression patterns with tSNE analysis (Figure 1A).

Survival analysis demonstrated that the survival outcome of cluster 3 is significantly poorer than other clusters (Figure 1B), and cluster 1 had the longest overall survival (OS) with a 5-years survival ratio of over 60%. We next stratified gliomas in the other three cohorts with a naïve Bayes model trained using the TCGA dataset and found that cluster1 remained the gliomas with the best prognosis while the other two clusters had significantly worse survival outcomes (Supplementary Figure S2A). The expression level of 12 CRGs in the three clusters was depicted with a heatmap (Figure 1C). The differences among the three clusters suggested that FDX1 and SLC31A1 were associated with the more aggressive cluster3 (Figure 1D). PDHA1 and ATP7B were higher in less aggressive cluster1. The differences in the expression level of the other eight CRGs were given in Supplementary Figure S2B. Furthermore, representative immunohistochemical (IHC) staining for SLC31A1 and ATP7B in high- and low-grade glioma from the Human Protein Atlas (Pontén et al., 2008) (https://www.proteinatlas.org/) was utilized to validate the results (Supplementary Figures S2C, S2D). The results of IHC revealed that the expression level of SLC31A1 in high-grade glioma was significantly higher than in low-grade glioma, and ATP7B was lower in high-grade glioma, which was in line with the results from sequencing.

**FIGURE 2:**
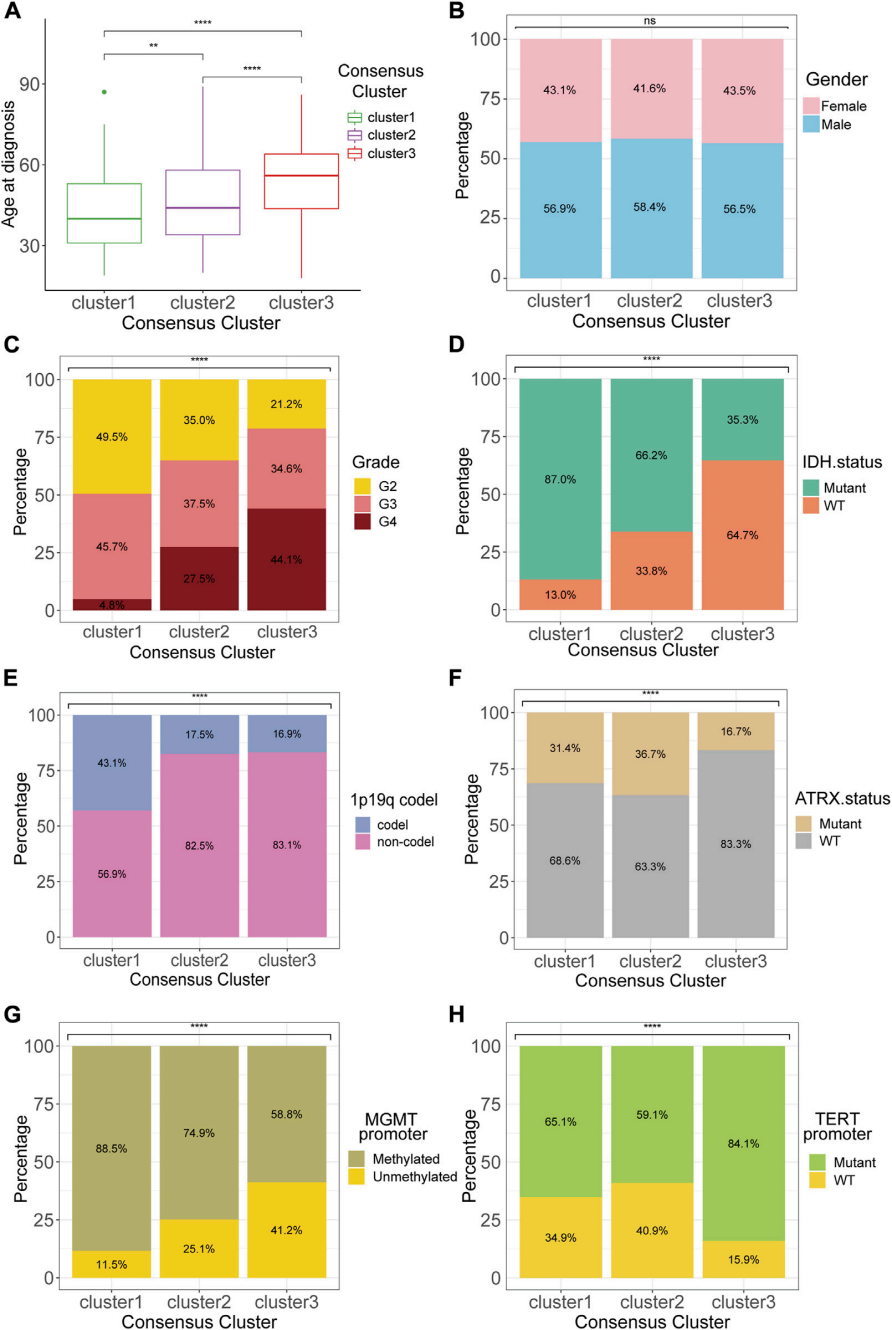
Clinicopathological features of the consensus clusters. Differences between consensus clusters in **(A)** age at diagnosis, **(B)** gender, **(C)** WHO grade, **(D)** IDH status, **(E)**1p19q codeletion, **(F)** ATRX status, **(G)** MGMT promoter methylation, and **(H)** TERT promoter mutation. *p < 0.05; **p < 0.01; ***p < 0.001; ****p < 0.0001.

Analyses of the clinical and pathological features among the three clusters revealed several noticeable trends. The patients with cluster 1 tumors, which presented with the best prognosis, had the smallest age at tumor diagnosis (Figure 2A). The sex ratios showed no significant difference between the three clusters (Figure 2B). Besides, the WHO grade 4 gliomas accounted for 44.1% of all tumors in cluster 3, which indicated cluster 3 gliomas had the highest WHO grade among all the three clusters, and this result was consistent with the results of survival analysis (Figure 2C). Isocitrate dehydrogenase (IDH) mutant, which emerged as an essential positive prognostic factor for gliomas, was detected in most (87.0%) patients of cluster 1 (Figure 2D). Moreover, the incidence of 1p/19q codeletion, recognized as “golden standard” for diagnosis of oligodendroglioma, was apparently more prevalent in cluster 1 compared to other clusters. Both alpha-thalassemia x-linked intellectual disability syndrome (ATRX) gene mutation and MGMT promoter methylation occurred less frequently in cluster 3 than in other clusters (Figures 2F,G). The highest incidence of telomerase reverse transcriptase (TERT) promoter mutation, a vital factor for both diagnosis and prognosis, was also observed in cluster 3 (Figure 2H).

**FIGURE 3:**
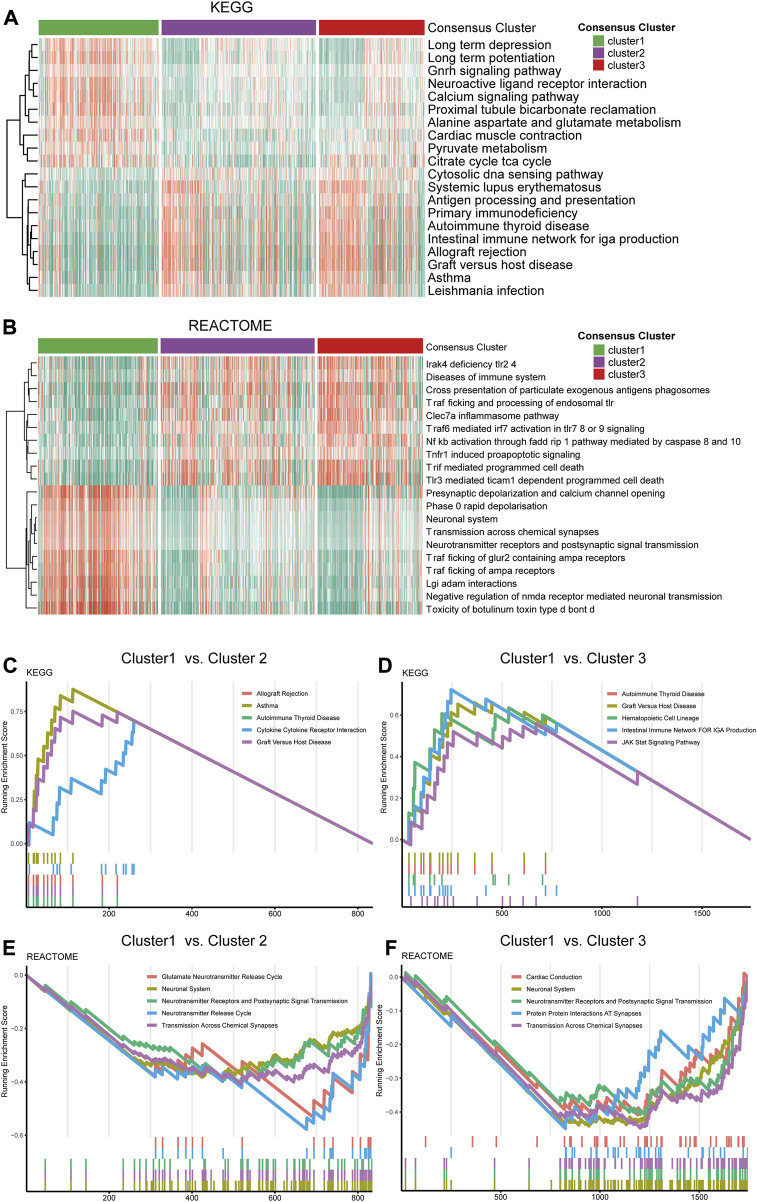
Functional analysis of the transcriptome of the consensus clusters. **(A)** Top 20 differentially expressed KEGG gene sets. **(B)** Top 20 differentially expressed REACTOME gene sets. **(C)** Top five pathways with the highest normalized enrichment score in the KEGG gene sets between cluster1 and cluster2, **(D)** between cluster1 and cluster3. **(E)** Top five pathways with the highest NES in the REACTOME gene sets between cluster1 and cluster2, **(F)** between cluster1 and cluster 3.

The GSVA analysis of the three clusters found distinctive pathway alternations. A number of pathways, including calcium signaling, pyruvate metabolism, neuroactive ligand receptor interaction, and GnRH signaling pathway, were significantly upregulated in cluster 1 compared to cluster 2 and 3. On the contrary, other pathways, including antigen processing and presentation, cytosolic DNA sensing pathway, and primary immunodeficiency, were more over-activated in cluster 2 and 3 than in cluster 1 (Figure 3A). Additionally, the REACTOME pathway gene sets were also utilized in enrichment analysis to explore the different pathway patterns of the three clusters (Figure 3B). The cytokine receptor interaction (normalized enrichment score (NES) = 2.908, adjusted *p*-value = 0.008), and graft versus host (NES = 3.128, adjusted *p*-value = 0.008) were ranked in the top five gene sets of the KEGG in the comparison between cluster 1 and 2 using GSEA (Figure 3C). Simultaneously, other top 5 enriched gene sets enriched in the DEGs between cluster 1 and 2, or cluster 1 and 3 in KEGG and REACTOME datasets were illustrated (Figures 3C–F).

### Gene mutations and copy number variations in the three CRG clusters

**FIGURE 4:**
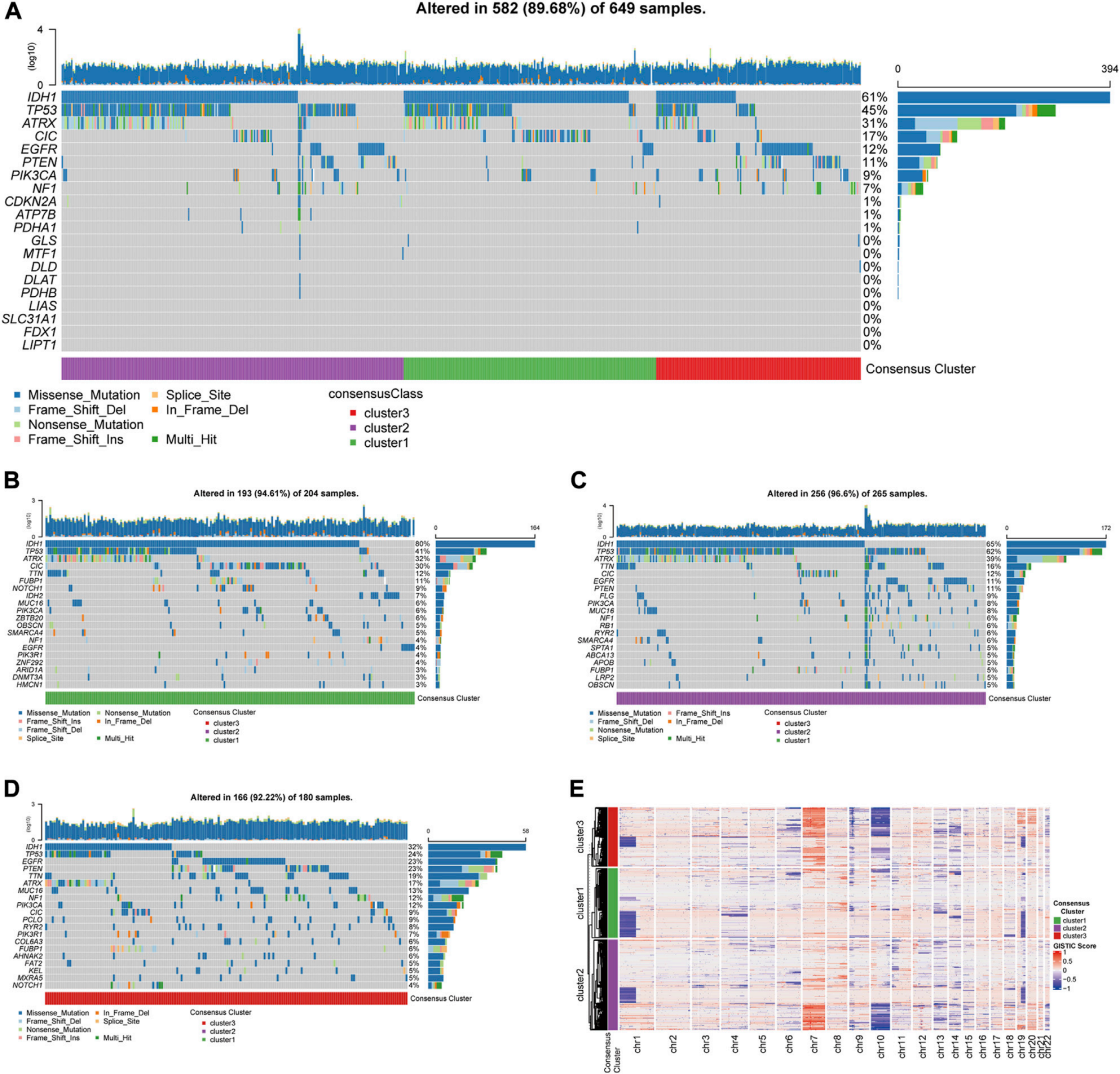
Genetic mutations and copy number variations of the three clusters. **(A)** Gene alternations of 12 CRGs and top 8 frequently mutated genes. **(B)** Top 20 mutated genes in cluster1, **(C)** in cluster 2, **(D)** in cluster 3. **(E)** Heatmap of copy number variations of the three clusters.

Analyses of the alternation rate of frequent mutations in glioma, including IDH1, TP53, ATRX, CIC, EGFR, PTEN, PIK3CA, and NF1, depicted the various patterns of genetic alternations among the three clusters (Figure 4A). Additionally, the 12 showed very low alternation rates, which rule out bias during expression analysis caused by gene alternations (Figure 4A). Besides, analysis of the top 20 frequently mutated genes in the three clusters revealed distinct mutational patterns between the three clusters, especially for cluster3, which was characterized by a less frequent mutation in the IDH genes but more frequent EGFR mutations (Figures 4B–D).

The analysis of copy number variations (CNVs) provided a differential karyotype landscape of each cluster (Figure 4E). The gain of chromosome 7 and the loss of chromosome 10 (+7/−10), a novel diagnostic marker of glioma associated with poor prognosis, were observed in most patients in cluster 3. On the contrary, the incidence of +7/−10 was remarkably lower in the subgroup with the best survival outcome, cluster 1.

### Differential immune characteristics of tumor microenvironment between the three CRG clusters

**FIGURE 5:**
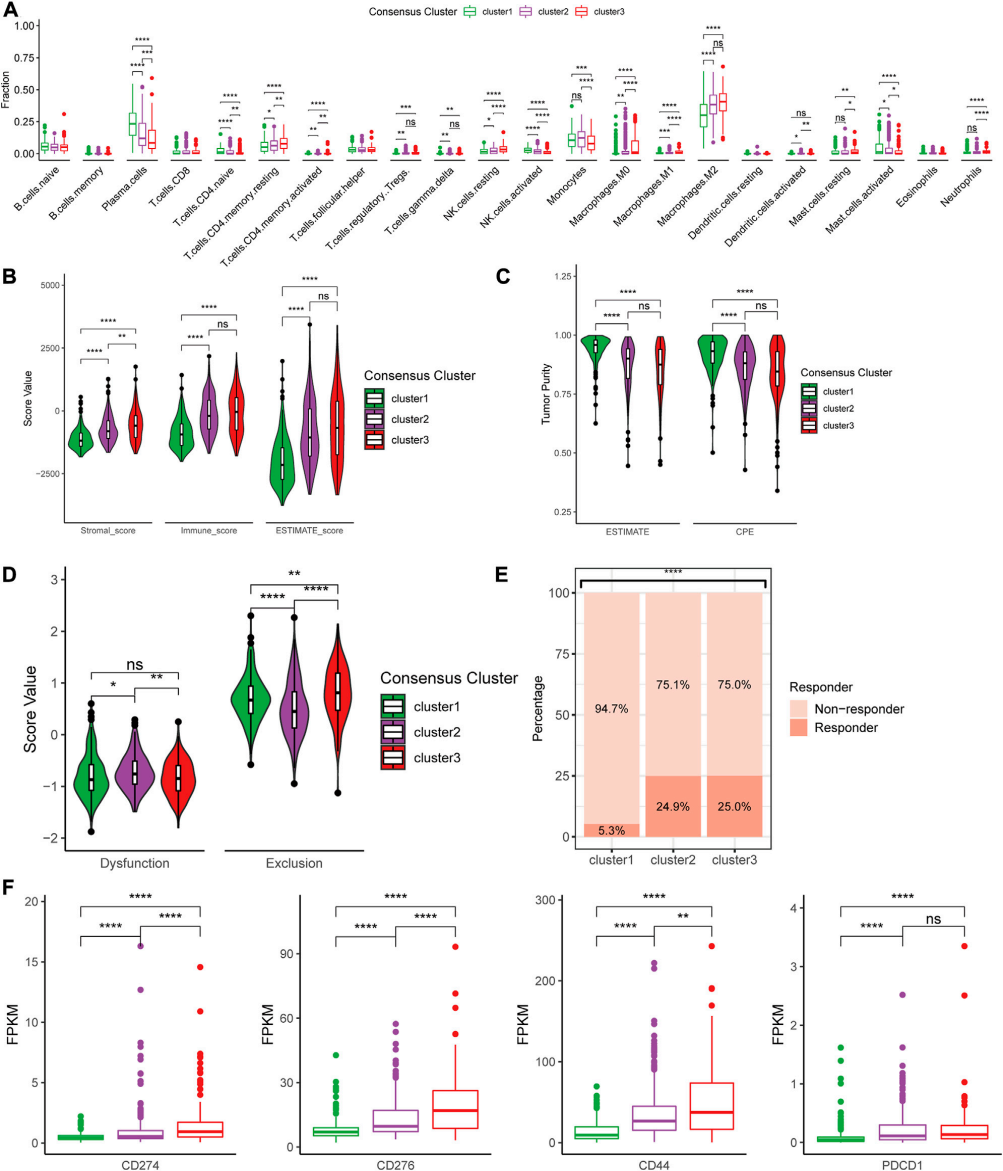
Differences in immune features of tumor microenvironment between the clusters. **(A)** Boxplot of the estimated fraction of 22 immune cells in tumors. **(B)** Stromal, immune, and ESTIMATE scores of the consensus clusters. **(C)** Tumor purity is calculated by the ESTIMATE and CPE algorithms. **(D)** T cell dysfunction and exclusion score of the consensus clusters. **(E)** Percentage of predicted responder to immune checkpoint inhibitor therapy in each consensus cluster. **(F)** The expression level of 33 immunotherapy-related genes in each consensus cluster. *p < 0.05; **p < 0.01; ***p < 0.001; ****p < 0.0001, post hoc Wilcoxon test results were shown if *p* < 0.05 in Kruskal–Wallis test.

Based on the three consensus clusters, we further conducted immune analyses to evaluate the relationships between cuproptosis and the immune microenvironment in glioma. The CIBERSORTx algorithm was utilized to predict the infiltration fraction of 22 immune cells in TME. In the estimation of CIBERSORTx, several immune cells, including CD8^+^ T cells, neutrophils, macrophages (M0, M1, and M2), and resting NK cells, were poorly recruited in cluster 1, whereas plasma cells were enriched in cluster 1 (Figure 5A). Additionally, the results of the ESTIMATE indicated that the stromal score, immune score, and ESTIMATE score were significantly lower in cluster 1 compared to other clusters (Figure 5B). Furthermore, the tumor purity decreased from cluster 1 to 3, opposite to immune scores (Figure 5C). These findings suggested that a higher tumor purity and lower immune scores may correlate positively with lower tumor aggressiveness.

Furthermore, we utilized the TIDE suite to explore the T cell dysfunction and exclusion scores (Figure 5D). Analysis of response to ICIs revealed that cluster 2 and 3 might show higher predicted response rates. Finally, to investigate the expression levels of multiple crucial genes in TME, differences in the expression of immune-related genes were analyzed between the three clusters. We found that several immunotherapy-related genes, including CD274 (PD-L1), CD276 (B7-H3), CD279 (PD-1), and CTLA4, were highly expressed in cluster 3 compared to other clusters (Figure 5F).

### Construction and validation of the cuproptosis-related genes risk signature and correlation with clinicopathological features

**FIGURE 6:**
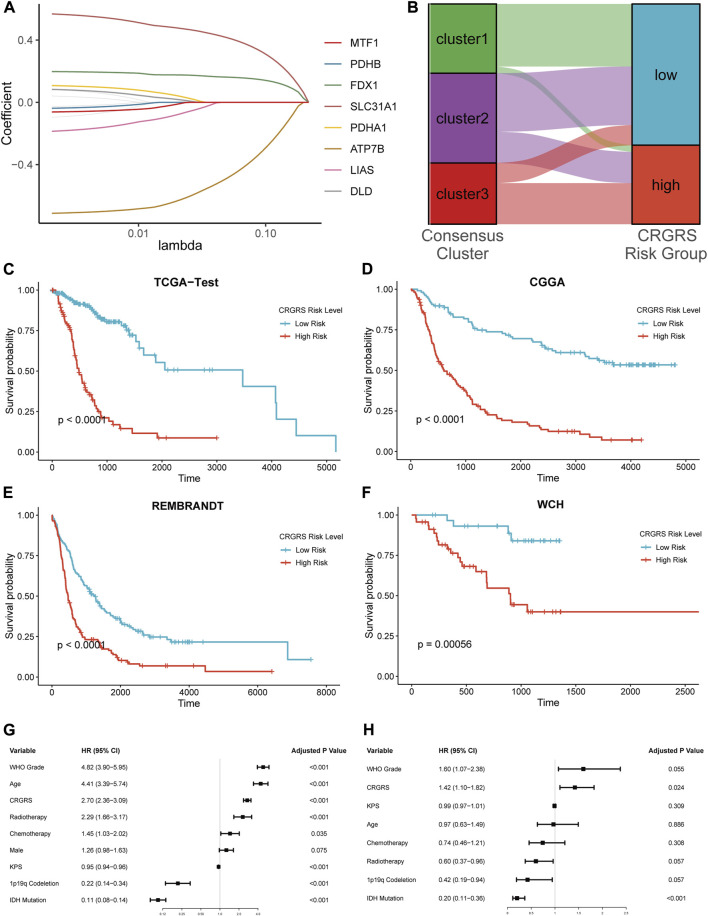
Expression signature of cuproptosis-related genes and its relationship with prognosis. **(A)** Average of coefficients of 7 critical CRGs in the LASSO Cox regression at each lambda value. **(B)** The relationship between the consensus clusters and two CRGRS risk groups. **(C)** K-M curve of the TCGA test set, cut off = 0.19. **(D)** K-M curve of the CGGA cohort, cut off = 0.39. **(E)** K-M curve of the REMBRANDT cohort, cut off = -9.64. **(F)** K-M curve of the WCH cohort, cut off = -0.056. **(G)** Univariate and **(H)** Multivariate Cox regression analysis of potential prognostic factors in overall survival of glioma.

To identify the genes for CRG risk signature (CRGRS) construction, we utilized the LASSO-Cox regression to filter the 12 CRGs with the training set data. Eventually, eight CRGs, MTF1, PDHB, FDX1, SLC31A1, PDHA1, ATP7B, LIAS, and DLD, were determined essential for constructing risk signature (Figure 6A). The formula for calculating the CRGRS was as follows:

0.152*SLC31A1+0.092*FDX1+0.024*PDHA1+0.013*DLD−0.016*PDHB−0.093*MTF1−0.147*LIAS−1.198*ATP7B

Next, we used the “surv_cutpoint” algorithm to find the optimal CRGRS cut-off and allocated the patients from each dataset into CRGRS low and high-risk subgroups. In the CRGRS risk grouping, most gliomas in cluster 1 were allocated to CRGRS low-risk group. Most of those in cluster 3 were allocated to the high-risk group (Figure 6B). The Kaplan-Meier survival curves confirmed that the patients with gliomas in the high-risk group had significantly poorer overall survival than the low-risk group in all four validation cohorts (Figures 6C–F).

To further evaluate the significance of the CRGRS in the clinical context, we first assessed its prognostic value using univariate followed by multivariate Cox analysis. The univariate Cox regression demonstrated that tumor grade, patient age, radiotherapy, chemotherapy, 1p19q codeletion, IDH mutation, and CRGRS emerged as significant univariate prognostic factors (Figure 6G). Further multivariate analysis proved that CRGRS was an independent prognostic factor (*p* = 0.024, HR: 1.42) after counterweighing the effects of other factors (Figure 6H). To demonstrate the added value of constructing the CRGRS, we also performed multivariate Cox regression analysis on each CRG with the prognostic clinicopathological factors, and found that none of the individual CRG were independent prognostic factor for glioma patients (Supplementary Table S1).

**FIGURE 7:**
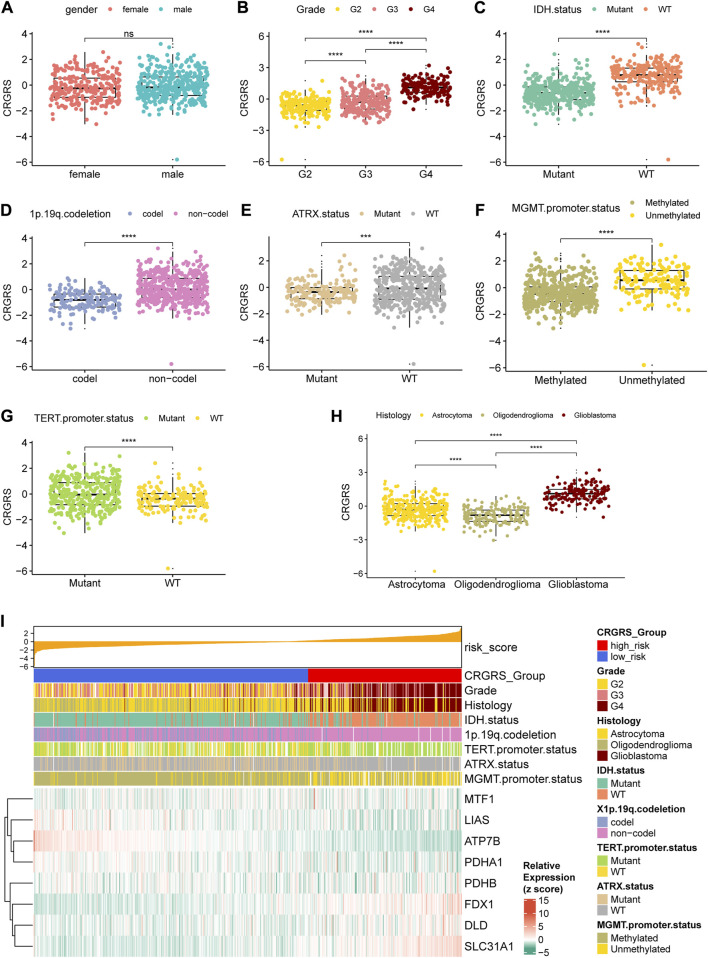
Relationship between clinicopathological features and the CRGRS. The differences in CRGRS with respect to **(A)** gender, **(B)** WHO grade, **(C)** IDH status, **(D)** 1p19q codeletion, **(E)** ATRX status, **(F)** MGMT promoter methylation, **(G)** TERT promoter mutation, **(H)** Histology diagnosis. **(I)** Heatmap sorted by CRGRS, elucidated clinicopathological features and expression of 7 critical CRGs in the TCGA cohort. *p < 0.05; **p < 0.01; ***p < 0.001; ****p < 0.0001.

In addition to the prognostic value, we also investigated the clinical implications of CRGRS by studying its association with other clinicopathological features. Between male and female groups, CRGRS was of no significant difference (Figure 7A). Furthermore, higher grade, IDH-wild type, non-1p19q codeletion, ATRX-wild type, MGMT unmethylated, TERT mutant, and glioblastoma were correlated with a higher CRGRS (Figures 7B–H). The heatmap combining CRGRS, clinicopathological features, and eight essential CRGs was used to illustrate their relationships (Figure 7I).

### Prediction of patient outcome with a CRGRS-based nomogram

**FIGURE 8:**
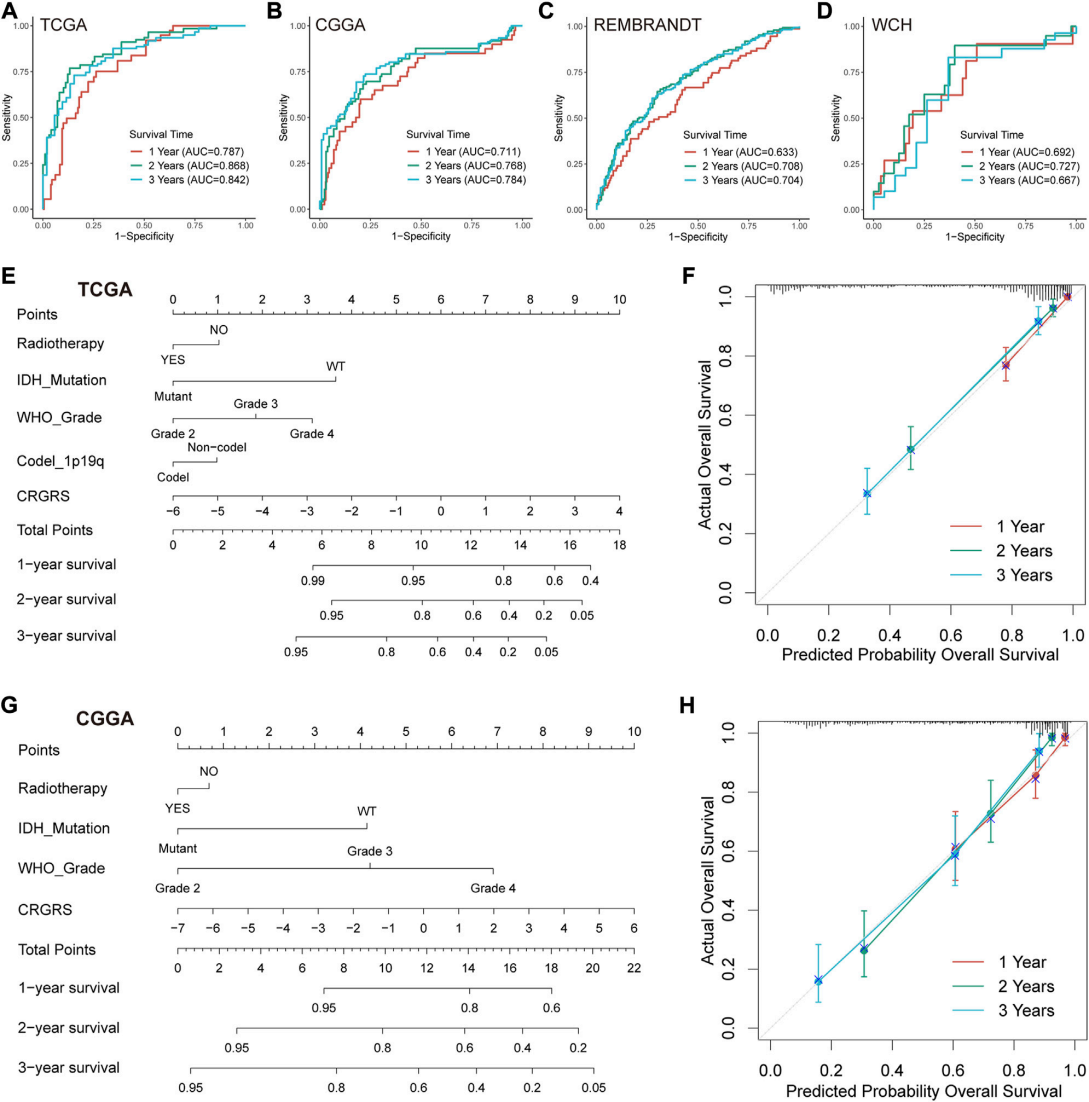
Prognostic value of CRGRS and construction of a CRGRS-based nomogram. ROC curves and matched AUC of 1-, 2-, and 3-years survival in **(A)**TCGA validation set, **(B)** CGGA cohort, **(C)** REMBRANDT cohort, and **(D)** WCH cohort. **(E)** Nomogram of 1-, 2-, 3-years survival of glioma patients based on **(E)** TCGA cohort, **(G)** CGGA cohort. Calibration plots of the nomogram based on **(F)** TCGA cohort and **(H)** CGGA cohort.

We first conducted ROC analyses to evaluate the performance of CRGRS alone in predicting patient survival at 1, 2, and 3 years. In the TCGA validation set, the AUCs of CRGRS were 0.787, 0.868, and 0.842 at 1, 2, and 3 years (Figure 8A), and similar performance was achieved in the other three validation cohorts (Figures 8B–D). We next integrate the CRGRS with other potential independent prognostic factors (*p* < 0.1) in the multivariate Cox regression analysis to construct a nomogram for personalized survival prediction based on the comprehensive clinical information for each patient. Finally, five factors, including IDH status, radiotherapy, grade, 1p19q codeletion, and CRGRS, were involved in nomogram construction (Figures 8E,G). The corrected C-index of the integrated nomogram was 0.818 for the TCGA cohort and 0.763 for the CGGA cohort. Moreover, the 1-, 2-, and 3-years calibration curves for the nomogram endorsed the accuracy of the survival time prediction (Figures 8F,H).

### Analyses of functional enrichment, genetic alternations, and immune characteristics of tumor microenvironment based on CRGRS risk groups

Based on the CRGRS, we conduct analyses of functional enrichment, gene mutations, CNVs, and immune characteristics to explore the differences between low and high-risk groups. GSEA of DEGs between the two risk groups discovered that cell cycle and cytokine receptor interaction were among the list of top 5 enriched KEGG gene sets (Supplementary Figure S3A), and hemostasis and innate/adaptive immune system were ranked within the top 5 of REACTOME gene sets (Supplementary Figure S3B). The GSVA analysis identified multiple gene sets differentially expressed between the risk groups, such as DNA replication, glutathione metabolism, transports of nucleotide sugars, and meiotic recombination. (Supplementary Figures 3C–F). Moreover, the analysis of genetic alterations found very few alternations in the eight critical CRGs involved in CRGRS construction (Supplementary Figure S4A). The top 20 mutated genes in the high-risk group included TP53, PTEN, EGFR, and NF1 (Supplementary Figure S4C), while those of the low-risk group were apparently different (Supplementary Figure S4B). Results of the CNVs analysis found an enormously higher incidence of +7/-10 chromosomes in the high-risk group (Supplementary Figure S4D).

**FIGURE 9:**
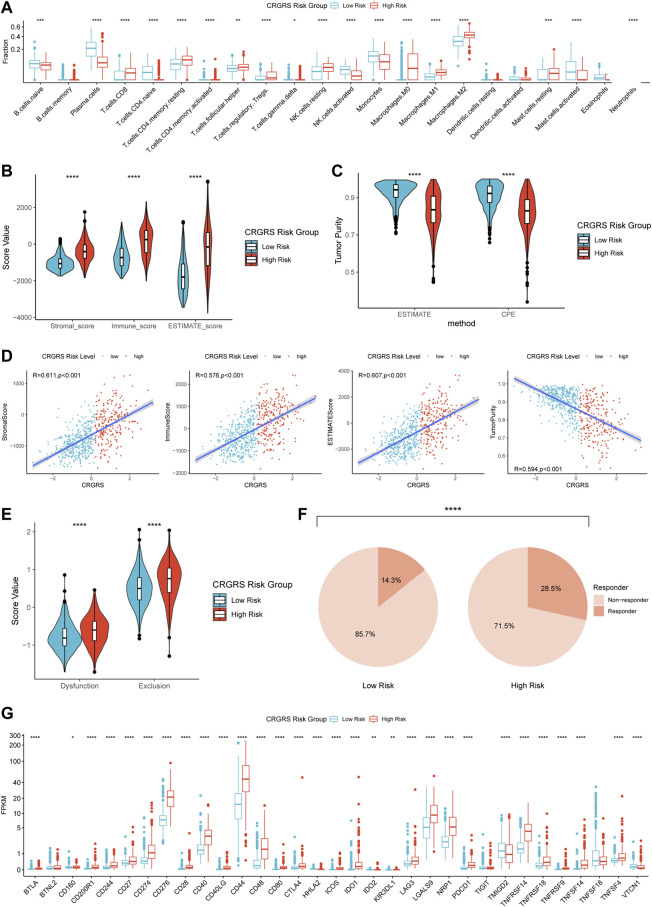
Differences in immune features of tumor microenvironment between two CRGRS risk groups. **(A)** Boxplot of the estimated fraction of 22 immune cells in tumors. **(B)** Stromal, immune, and ESTIMATE scores of the two risk groups. **(C)** Tumor purity of the two risk groups based on the ESTIMATE and CPE algorithms. **(D)** Analyses of correlations of CRGRS with the stromal, immune, ESTIMATE score, and tumor purity. **(E)** T cell dysfunction and exclusion score of two risk groups. **(F)** Percentage of predicted responders to immune checkpoint inhibitors therapy in each risk group. **(G)** The expression level of 33 immunotherapy-related genes in two risk groups. *p < 0.05; **p < 0.01; ***p < 0.001; ****p < 0.0001.

CIBERSORTx estimation of the immune cell fractions indicated different patterns of immune cell infiltration between the two risk groups. The predicted fractions of macrophages (M0, M1, M2), CD8^+^ T cells, regulatory T cells (Tregs), resting NK cells, and neutrophils were significantly lower in the low-risk group compared to the high-risk group (Figure 9A). On the contrary, naïve B cells, plasma cells, activated NK cells, and monocytes were more abundant in the low-risk group (Figure 9A). Analyses of the immune scores revealed that the low-risk group had a remarkably lower stromal score, immune score, and ESTIMATE score (Figure 9B), whereas the tumor purity of the low-risk group was significantly higher than that of the high-risk group (Figure 9C). The correlation analysis also proved that CRGRS positively correlated with the stromal, immune, and ESTIMATE scores but negatively correlated with the tumor purity (Figure 9D). The estimated T cell dysfunction and exclusion scores were higher in the high-risk group (Figure 9E). Prediction of immunotherapy response using TIDE indicated that patients with high-risk gliomas were more likely to benefit from ICIs (Figure 9F). Most of the tumor immunity-related genes, including PD-L1 (CD274), PD-1 (CD279), CTLA4, and B7H3 (CD276), were at a higher expression level in the high-risk group. Most of the findings about the immune characteristics of the tumor microenvironment could be validated in the CGGA, REMBRANDT, and our WCH cohort (Supplementary Figure S5).

## Discussion

By estimation, there are 308 thousand new cases of Central Nervous System (CNS) malignant tumors worldwide every year (Sung et al., 2021), of which 80% are gliomas (Ostrom et al., 2021). Despite persistent research efforts worldwide, the treatment outcome of gliomas, especially glioblastomas, remains unfavorable. Cancer immunotherapy, targeting enhancing natural defenses to attack malignant cells, has been confirmed to improve outcomes in multiple cancers (Eggermont et al., 2018; Gandhi et al., 2018; Choueiri et al., 2021; Cortes et al., 2022). Nevertheless, almost all immunotherapy attempts on glioblastoma failed to improve overall survival (Weller et al., 2017; Wakabayashi et al., 2018; Reardon et al., 2020; Lim et al., 2022; Omuro et al., 2022). The immunologically quiescent environment of CNS resulting from the blood-brain barrier (BBB) could be a potential reason for the failures of immunotherapy (Jackson et al., 2019). However, a newly-discovered unique lymphatic pathway paralleling the dural venous sinuses may provide a channel for the antigen-presenting cells to egress from the brain (Louveau et al., 2015), prime B and T lymphocytes, and then evoke robust immune responses (Lim et al., 2018), which could explain immune activities in brain abscesses and multiple sclerosis (Waksman and Adams, 1955; Canessa et al., 1992). These refreshed insights into the distinct immunological pathways in CNS disease suggest that there is still an abundance of opportunities and challenges for immunotherapy application in gliomas.

Copper has been proved to be involved in cell proliferation and death pathways (Ge et al., 2022). As an essential cofactor for multiple enzymes, copper mediates several cell functions, including antioxidant defense, synthesis of hormones, and respiration of mitochondria (Solomon et al., 1996; Que et al., 2008). A recent study characterized the cuproptosis pathway, which defined a copper-dependent RCD mediated by the binding of copper to lipoylated components of the Krebs cycle (Tsvetkov et al., 2022). This novel form of RCDs became a potential target for glioma treatment to overturn the failures of multiple novel therapies caused by apoptosis resistance (Gong et al., 2019). To understand if cuproptosis is involved in the pathophysiology of gliomas and influence the immune characteristics of the glioma microenvironment, we investigated the expression pattern of cuproptosis-related genes (CRG) in gliomas and associated the CRG expression signature with the clinical, molecular, and immunological landscape of gliomas using publicly available and in-house dataset.

In our present study, we first cluster the glioma patients into three subgroups based on the different expression patterns of twelve cuproptosis-related genes. The patterns of clinicopathological characteristics and survival outcomes are diverse in these clusters. Several critical prognostic factors of glioma, including IDH mutation, 1p/19q codeletion, and TERT promoter mutation (Eckel-Passow et al., 2015), were found to be tightly correlated with the expression pattern of CRGs and the CRGRS. Of note, isocitrate dehydrogenase (IDH) was an essential enzyme of the tricarboxylic acid (TCA) cycle, whose mutations lead to aberrant tricarboxylic acid cycle and producing oncometabolite D-2-Hydroxyglutarate (D-2HG) (Dang et al., 2009; Bleeker et al., 2010). Since the cuproptosis mechanism is known to interact with the TCA, it was no surprise to us that the CRGRS was closely associated with the IDH mutation (Que et al., 2008; Tsvetkov et al., 2022). Besides, the functional gene set analysis results suggested that different expression patterns of CRGs were involved in regulating the citrate cycle, confirming the relationship between cuproptosis and the TCA cycle. The incidence of different gene alterations differs widely among clusters. For example, the epidermal growth factor receptor (EGFR), which has been implicated in glioma development (Eskilsson et al., 2018), is frequently amplified, mutated, and overexpressed in malignant gliomas, especially glioblastoma (Brennan et al., 2013). Multiple EGFR-targeted therapies have succeeded in NSCLC (Mok et al., 2009; Ramalingam et al., 2020). However, the EGFR inhibitors failed to improve overall survival in glioblastoma patients (Chinot et al., 2014; Gilbert et al., 2014; Weller et al., 2017). The heterogeneity of EGFRs in glioma might be an essential reason for the failure of anti-EGFR therapy in glioma. Gene alternation analysis of different clusters shows that the incidence of EGFR alternations variates largely. The cluster with the best prognosis hardly harbors EGFR alternations. Nevertheless, the rate surges to 23% in the most aggressive cluster. For further validation, this incidence of CRGRS high-risk group was 26%, remarkably higher than the low-risk group’s 4%. These results indicated that cuproptosis was tightly related to EGFR status. Although the relationship between cuproptosis and EGFR remained unclear, more studies in this field might provide a novel direction for EGFR inhibitors’ application in glioma and overturn previous failures.

The cooperation of copper importer SLC31A1 (CTR1) and the copper exporter ATP7A and ATP7B is essential for maintaining the intracellular copper concentration (Lutsenko, 2010). After overexpressing the copper importer SLC31A1, cells’ sensitivity to copper concentration surged dramatically (Tsvetkov et al., 2022). Deleting the copper exporter ATP7B would lead to intracellular copper accumulation and cell death (Lutsenko, 2008; Muchenditsi et al., 2021). Our result showed that the high expression level of SLC31A1 and low expression of ATP7B were found in more aggressive gliomas, which indicate high influx and low efflux of copper, resulting in copper retention in these tumors. FDX1 also reduces Cu^2+^ to Cu^+^ and contributes to cuproptosis sensitivity by enhancing lipoylation of TCA carbon entry regulators, including DLAT (Tsvetkov et al., 2019; Tsvetkov et al., 2022), was also expressed at a higher level in the high CRGRS group. Together, these results suggest higher cuproptosis potential in the high CRGRS gliomas. However, the conclusions on the phenotype of cuproptosis should be interpreted cautiously, considering the complex interactions between the altered metabolism program in gliomas. For instance, malignant gliomas are known to produce abundant glutathione (GSH), which could block cuproptosis by chelating copper (Rocha et al., 2014; Tsvetkov et al., 2022). Nevertheless, as suggested by our results, the mechanism of cuproptosis appears to be an attractive therapeutic target to exploit for malignant gliomas.

The immune analysis in the present study evaluated the relationship between cuproptosis and the tumor immune microenvironment in glioma. The CIBERSORTx analysis estimated a more abundant infiltration of multiple immune cells in the high-risk group. Notably, M2 macrophages, which are recognized for a critical role in immunosuppressing and tumor promotion (Noy and Pollard, 2014), are enriched in the glioma of the high-risk group. Previous studies demonstrated that malignant gliomas were significantly more capable of recruiting blood-derived TAMs than low-grade glioma, but the number of microglial-related TAMs shows no significance between the two glioma subtypes (Müller et al., 2017). TAMs play a crucial role in secreting chemokines to recruit Treg cells, producing cytokines to suppress functions of T cells, and upregulating immunosuppressive surface proteins (Curiel et al., 2004; Colombo and Piconese, 2007; Yang and Zhang, 2017). The results of these studies corroborate the abundant infiltration of Treg cells in the high-risk group found in the present study.

In addition to the cellular crosstalk, TAMs often express PD-L1 to inhibit phagocytosis and tumor immunity (Gordon et al., 2017), which was a consensual target for ICIs to enhance anti-tumor immunity (Cha et al., 2019). The overexpression of CD274 (encoding PD-L1) was also confirmed in the high-risk group. These results demonstrate the potential of CRGRS to predict the immune characteristics of the tumor microenvironment in glioma. Those gliomas in CRGRS high-risk group would harbor more immune cell infiltration, express more immunotherapy targets (including PD-1, PD-L1, CTLA4, and B7H3), and therefore could potentially present a better response to immunotherapy. Glioma is often considered an immunologically cold tumor, which is a fundamental reason for the failures of immunotherapy. Only lesser than 3% of cells express PD-L1 in glioblastoma (Nduom et al., 2016). Predicting immune characteristics with CRGRS would help when choosing an optimal immunotherapy strategy.

Our study comprehensively explored the relationship of cuproptosis-related genes with the clinicopathological features, prognosis, and immune characteristics of glioma. However, there are still several limitations to our study. First, due to the usage of four independent datasets, the sequencing protocols and data preprocessing procedure varied for each dataset. Second, the data from the REMBRANDT cohort lacks some essential markers, such as IDH mutation. Moreover, although we thoroughly validated our results using multiple independent validation datasets and an in-house dataset, the findings of the present study and the mechanism for the associations still require experimental validation and exploration. Finally, the role of cuproptosis in glioma represents a promising research target and needs to be further elucidated by future studies.

## Conclusion

In conclusion, based on the comprehensive analyses of four datasets, we demonstrated that the expression of cuproptosis-related genes was tightly correlated with clinicopathological features, overall survival, and tumor immune microenvironment of glioma. The novel cuproptosis-related genes risk signature achieved favorable accuracy in predicting prognosis in glioma patients. Furthermore, gliomas with high CRGRS risk potentially harbored more abundant immune cell infiltration and expressed immunotherapy targets at a higher level. Hence, the CRGRS may be utilized to guide the application of immunotherapy in glioma.

## Data Availability

The sequencing data of West China Hospital generated in this study are available at the Genome Sequence Archive for Humans: accession code HRA002839 (access link: https://ngdc.cncb.ac.cn/gsa-human/s/JQssVoV1).
