# Supplementary material for: Prognostic value of cuproptosis-related genes signature and its impact on the reshaped immune microenvironment of glioma
Source: Front Pharmacol. 2022 Oct 4;13:1016520. doi: 10.3389/fphar.2022.1016520 (PMC9576857; doi:10.3389/fphar.2022.1016520)
Supplement: Supplementary file 1 [file Table1.DOCX]

**Supplementary table 1. Multivariate Cox regression analysis on each CRG with the prognostic clinicopathological factors.**

|  | **HR** | **P-value** | **Adjusted P-value** |
| --- | --- | --- | --- |
| **SLC31A1** | 1.337 (CI: 1.066 - 1.676) | 0.012 | 0.142 |
| **LIPT1** | 0.818 (CI: 0.690 - 0.970) | 0.021 | 0.231 |
| **DLAT** | 1.238 (CI: 1.028 - 1.492) | 0.024 | 0.244 |
| **CDKN2A** | 0.889 (CI: 0.795 - 0.993) | 0.037 | 0.334 |
| **LIAS** | 0.839 (CI: 0.697 - 1.011) | 0.065 | 0.519 |
| **FDX1** | 1.165 (CI: 0.977 - 1.389) | 0.088 | 0.618 |
| **MTF1** | 1.180 (CI: 0.973 - 1.432) | 0.093 | 0.618 |
| **ATP7B** | 0.853 (CI: 0.663 - 1.097) | 0.216 | 1.000 |
| **PDHA1** | 0.918 (CI: 0.758 - 1.113) | 0.386 | 1.000 |
| **PDHB** | 1.044 (CI: 0.877 - 1.242) | 0.629 | 1.000 |
| **GLS** | 1.037 (CI: 0.819 - 1.312) | 0.763 | 1.000 |
| **DLD** | 0.974 (CI: 0.821 - 1.156) | 0.764 | 1.000 |
