# Supplementary figures and images for: Prognostic value of cuproptosis-related genes signature and its impact on the reshaped immune microenvironment of glioma
Source: Front Pharmacol. 2022 Oct 4;13:1016520. doi: 10.3389/fphar.2022.1016520 (PMC9576857; doi:10.3389/fphar.2022.1016520)

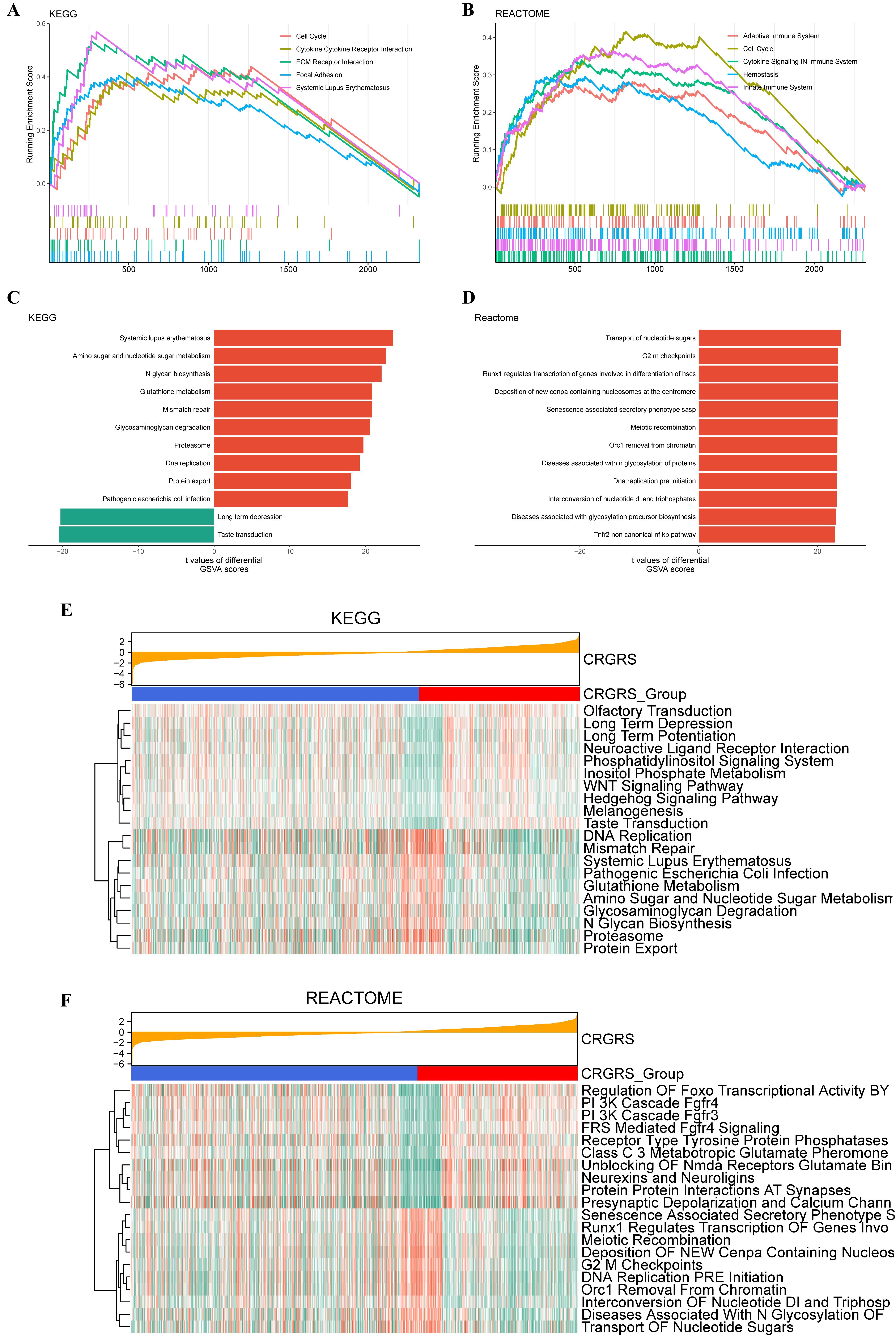

Supplement: Supplementary file 2 [file Image3.TIF]

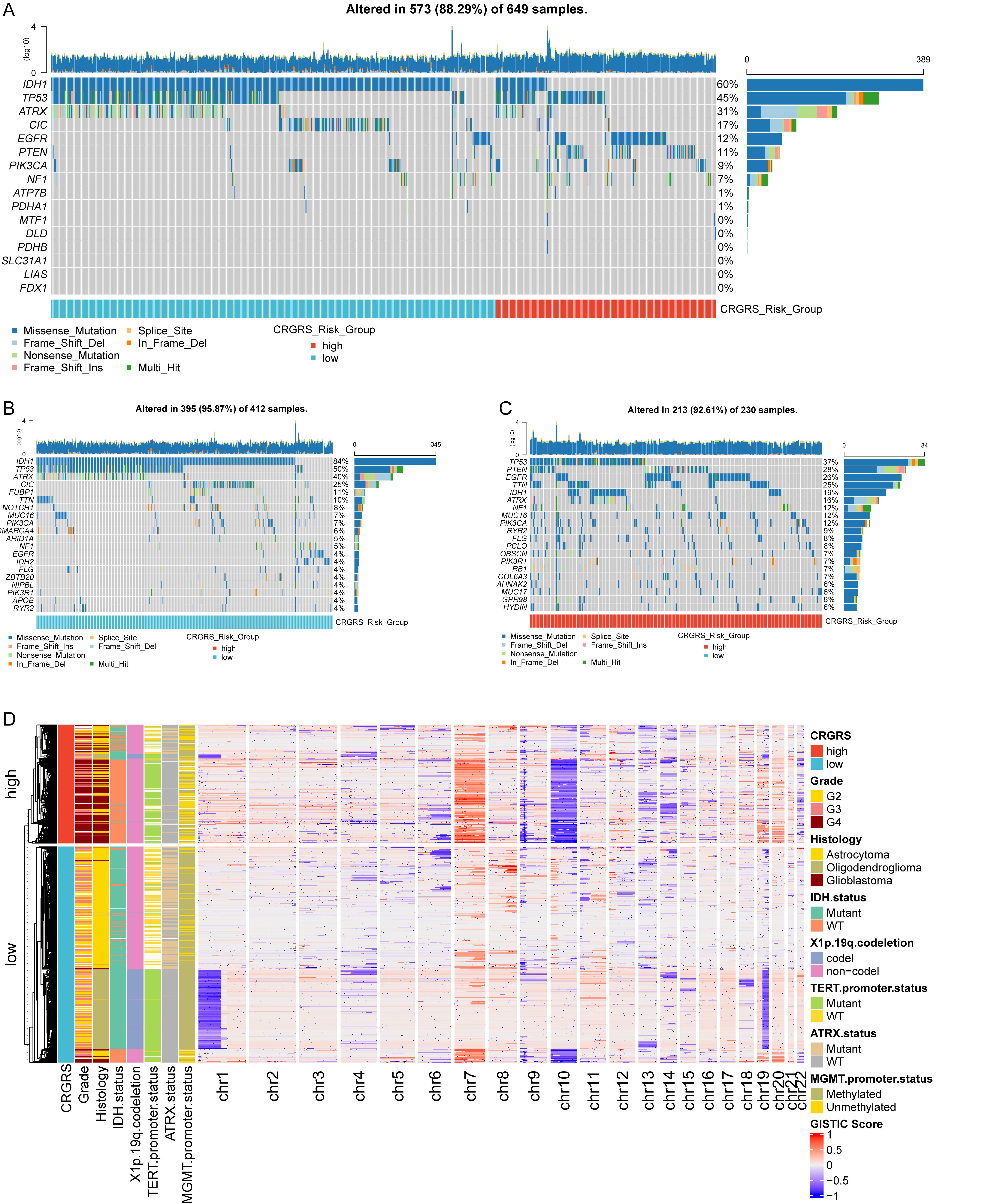

Supplement: Supplementary file 3 [file Image4.TIF]

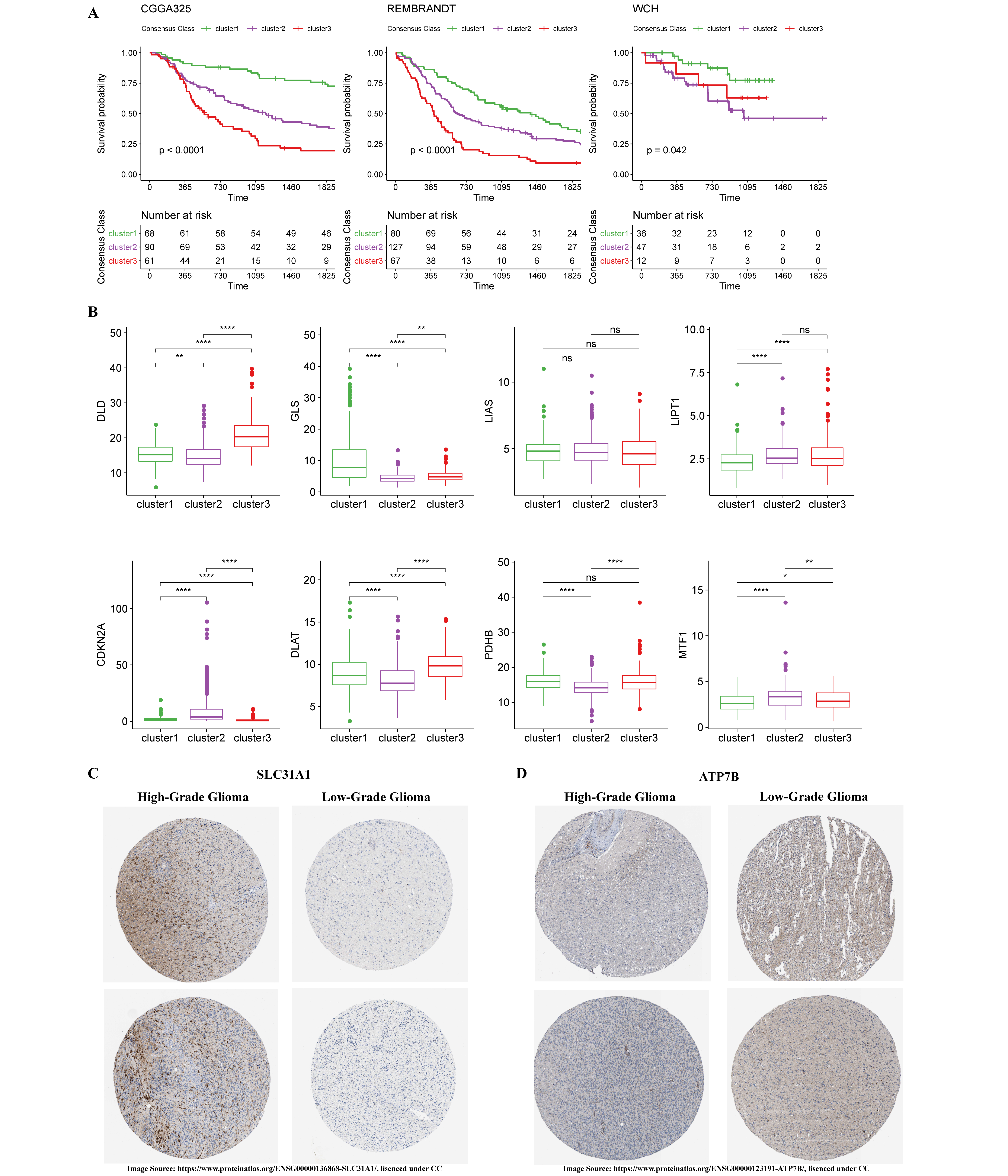

Supplement: Supplementary file 4 [file Image2.TIF]

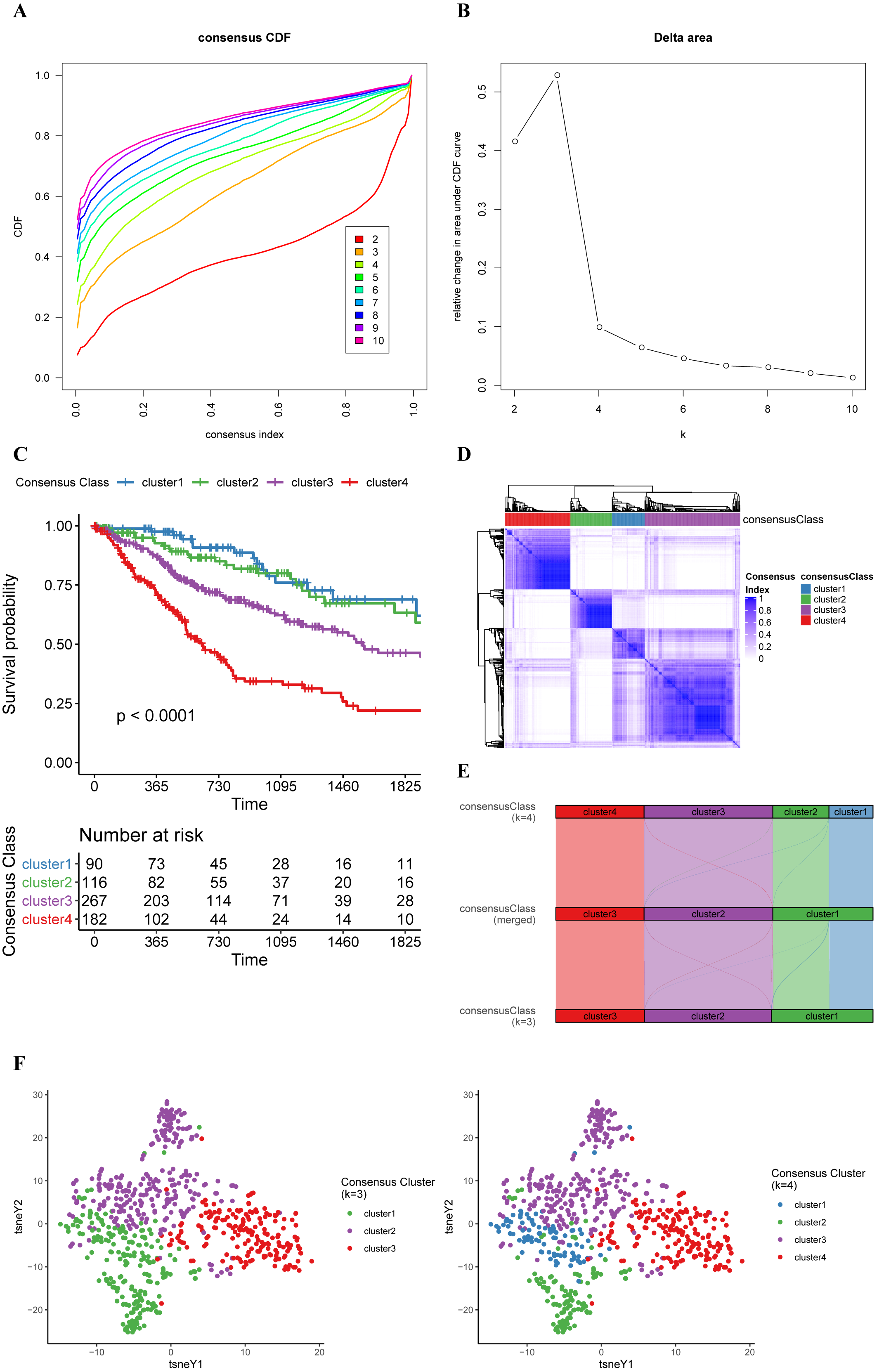

Supplement: Supplementary file 5 [file Image1.TIF]
